# Modulation of heme and tumor vascular oxygenation- a novel strategy for lung cancer therapy

**DOI:** 10.18632/oncoscience.569

**Published:** 2022-12-02

**Authors:** Poorva Ghosh, Ralph P. Mason, Li Liu, Li Zhang

**Affiliations:** ^1^Department of Biological Sciences, University of Texas at Dallas, Richardson, TX 75080, USA; ^2^Corvus Pharmaceuticals Inc., Burlingame, CA 94010, USA; ^3^Department of Radiology, The University of Texas Southwestern Medical Center, Dallas, TX 75235, USA

**Keywords:** heme, tumor vascular oxygenation, lung cancer

## Abstract

Hypoxia and faulty vasculature are well-known hallmarks of cancer and in addition to being associated with poor prognosis in patients, these hallmarks are also known to contribute to therapy resistance. In recent years, therapeutics that alleviate hypoxia and promote normalization of vasculature are being explored for cancer therapy. In addition to being hypoxic, cancers such as non-small cell lung cancers exhibit elevated oxidative phosphorylation. Therapeutic strategies that can normalize vasculature and reduce oxidative phosphorylation could greatly benefit the landscape of cancer therapeutics. Here, we highlight a heme-targeting therapeutic strategy that demonstrates significant tumor growth inhibition in non-small cell lung cancer mouse models using multi-spectral optoacoustic tomography.

The tumor microenvironment (TME) is often highly heterogeneous and influences invasion, metastasis, and cancer progression as well as response to many types of therapy. Tumor vasculature and oxygenation are important aspects of TME and faulty tumor vascular oxygenation has been identified as a marker for poor prognosis in cancer.

Hypoxia arises from poor oxygen supply due to inefficient vasculature as well as increased oxygen demand due to rapid growth. Hypoxia is associated with poor prognosis, increased genomic instability, elevated metastatic potential, and resistance to chemotherapy and radiotherapy [1]. Hypoxia and induction of angiogenesis are prognostic markers of cancer and are associated with therapy resistance. Neovasculature generation by angiogenesis occurs in tumors to fulfill nutrition and oxygenation needs as well as providing means to remove metabolic waste and carbon dioxide [2]. However, this neovasculature is poorly functional. Such tumor vessels are characterized by reduced blood flow, endothelial cell sprouting, disruption of endothelial cell junctions, loss of pericytes coverage, and increased leakiness. This results in increased hypoxia and intravasation of tumor cells [3]. The tumor microenvironment is not only hypoxic and acidic, but is also surrounded by high interstitial pressure which hinders drug delivery into tumors [4–6]. Therefore, anti-angiogenic therapies have been investigated extensively and have focused on inhibiting new vessel formation or selective destruction of the existing tumor vessels to starve tumor cells [7–9]. To overcome challenges associated with drug delivery due to faulty vasculature in tumors, strategies to normalize vasculature are being explored [10]. Normalized tumor vasculature leads to decreased leakage, increased perfusion, and reduced hypoxia, which improves the whole tumor microenvironment to make it favorable for drug delivery and overcome therapy resistance [11].

Cancer cells exhibit upregulated glycolysis, which has led to the assumption that oxidative phosphorylation (OXPHOS) is downregulated in all cancers. However, there is increasing evidence of elevated levels of OXPHOS in many types of cancer [12–14]. Certain types of NSCLCs (Non-small cell lung cancers) are found to be heavily reliant on OXPHOS [15]. NSCLCs also exhibit metabolic heterogeneity within tumors [14]. In these cancers, inhibition of OXPHOS can provide an effective therapeutic strategy. Reduced oxygen availability in hypoxic regions of tumors may not limit OXPHOS [12], since ATP is known to be generated by OXPHOS in tumors even at very low oxygen tensions [16]. Therefore, targeting OXPHOS could be an effective way to reduce the consumption of oxygen and increase oxygen availability in the tissue. This results in increased oxygen diffusion into otherwise hypoxic tumor regions thereby alleviating tumor hypoxia. Studies have shown that reduction in the OCR (Oxygen Consumption rate) can alleviate the central region of hypoxia by increasing the availability of free oxygen [17, 18]. Indeed, modeling has shown that reduced oxygen consumption can be more effective than increased oxygen delivery [19].

There is overwhelming evidence pointing to the reliance of cancer cells on mitochondrial dysfunction and oxidative metabolism for their growth and progression. This suggests that targeting OXPHOS and mitochondria, in general, can be an effective strategy to treat cancer. However, to treat genetically and metabolically diverse cancers, it is essential to investigate novel therapeutic avenues that would be effective against a wide variety of cancers. Targeting OXPHOS via limitation of heme is one such promising approach [15, 20].

Heme is central to oxygen utilization and serves as a prosthetic group or cofactor for many OXPHOS proteins. HSP2 or HeSP2 (heme-sequestering peptide 2) and CycT (cyclopamine tartrate) are two heme targeting agents that effectively inhibit OXPHOS, and have been shown to suppress lung tumor growth and progression in human tumor xenograft mouse models [15, 20] (Figure 1). HSP2 (generated from bacterial hemophore HasA *Y. pestis*) binds to heme strongly, inhibits heme uptake, decreases mitochondrial heme levels, and diminishes OXPHOS and ATP generation in NSCLC cells. HSP2 has significantly suppressed subcutaneous and orthotopic NSCLC tumor xenografts in mice [15]. Many imaging tools are available for non-invasive assessment of tumor oxygenation [21] and Ghosh et al. effectively used multispectral optoacoustic tomography (MSOT) and oxygen-enhanced (OE) MSOT to monitor changes in tumor vasculature and oxygenation in live animals. They demonstrated that HSP2 and CycT, which also inhibit OXPHOS and oxygen consumption not only reduce ATP generation, but also alleviate tumor hypoxia and normalize tumor vasculature in NSCLC tumors [22] (Figure 1).

**Figure 1:**
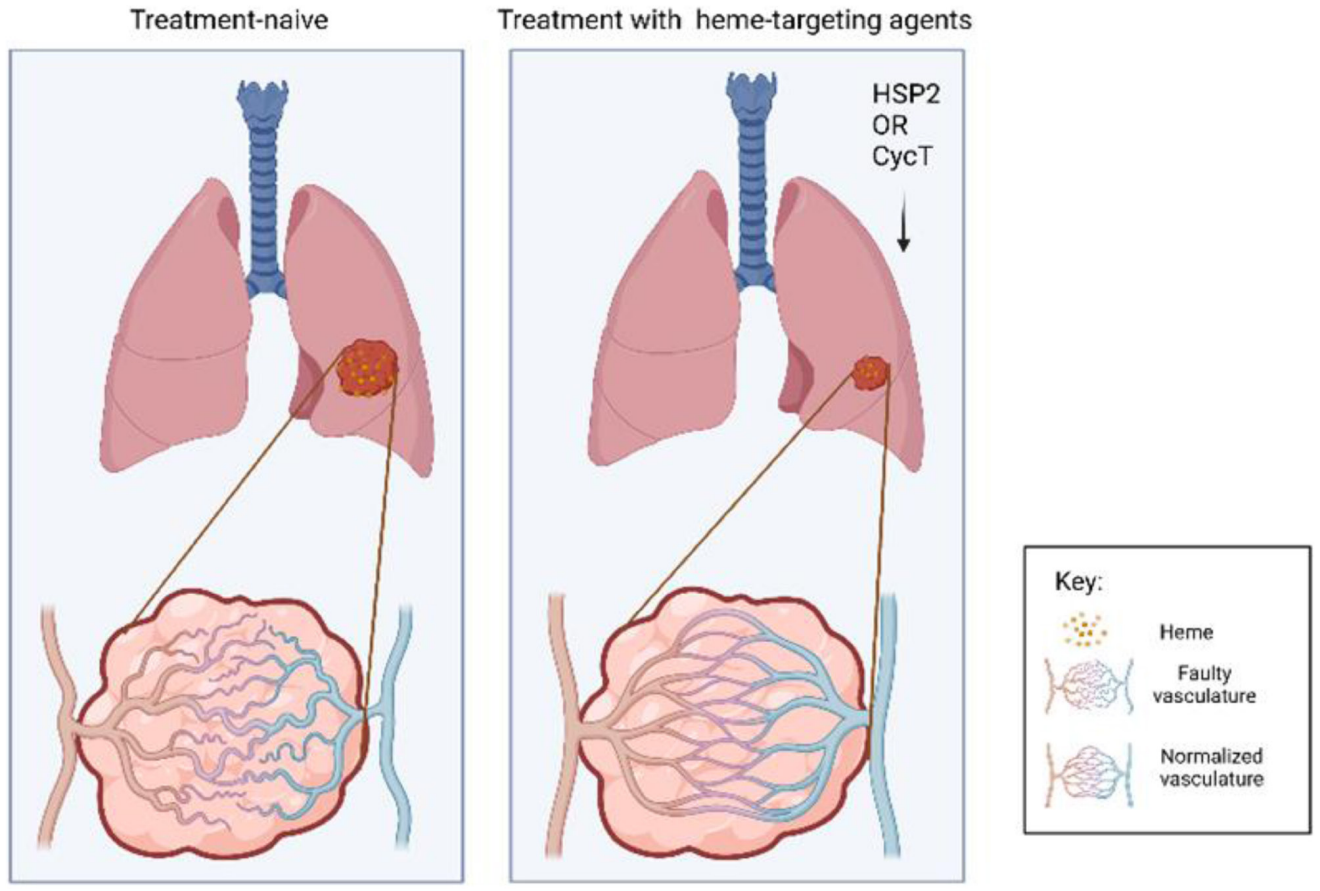
Heme targeting drugs improve tumor vascular oxygenation in NSCLC tumors. NSCLC tumors have elevated heme flux and OXPHOS along with increased hypoxia with faulty vasculature (panel on the left). Treatment with heme-targeting drugs like HSP2 and CycT leads to lower heme flux, decrease in OXPHOS which subsequently leads to alleviation of hypoxia and normalization of vasculature (panel on the right).

These heme-targeting drugs are effective at reducing tumor burden in autochthonous mouse models of lung cancer [23]. Heme-targeting drugs are potentially promising therapeutics in other cancers in addition to NSCLC [24]. Dynamic monitoring of the tumor microenvironment using optoacoustic tomography offers a valuable tool to determine time of treatment administration and asses its efficacy. The use of heme-targeting drugs to modulate the tumor microenvironment along with monitoring of vascular oxygenation status using optoacoustic tomography could be a promising approach to effectively inhibit angiogenesis, normalize vasculature, and alleviate hypoxia in tumors. This, combined with the current standard of care therapies could be a highly effective approach to target a wide variety of cancers and could significantly improve patient outcome.
